# Change Blindness Is Influenced by Both Contrast Energy and Subjective Importance within Local Regions of the Image

**DOI:** 10.3389/fpsyg.2017.01718

**Published:** 2017-10-04

**Authors:** Wietske Zuiderbaan, Jonathan van Leeuwen, Serge O. Dumoulin

**Affiliations:** ^1^Department of Experimental Psychology, Helmholtz Institute, Utrecht University, Utrecht, Netherlands; ^2^Department of Experimental and Applied Psychology, Vrije Universiteit Amsterdam, Amsterdam, Netherlands; ^3^Spinoza Centre for Neuroimaging, Amsterdam, Netherlands

**Keywords:** change detection, contrast energy, subjective importance, natural images, scene perception

## Abstract

Our visual system receives an enormous amount of information, but not all information is retained. This is exemplified by the fact that subjects fail to detect large changes in a visual scene, i.e., change-blindness. Current theories propose that our ability to detect these changes is influenced by the gist or interpretation of an image. On the other hand, stimulus-driven image features such as contrast energy dominate the representation in early visual cortex (De Valois and De Valois, 1988; Boynton et al., 1999; Olman et al., 2004; Mante and Carandini, 2005; Dumoulin et al., 2008). Here we investigated whether contrast energy contributes to our ability to detect changes within a visual scene. We compared the ability to detect changes in contrast energy together with changes to a measure of the interpretation of an image. We used subjective important aspects of the image as a measure of the interpretation of an image. We measured reaction times while manipulating the contrast energy and subjective important properties using the change blindness paradigm. Our results suggest that our ability to detect changes in a visual scene is not only influenced by the subjective importance, but also by contrast energy. Also, we find that contrast energy and subjective importance interact. We speculate that contrast energy and subjective important properties are not independently represented in the visual system. Thus, our results suggest that the information that is retained of a visual scene is both influenced by stimulus-driven information as well as the interpretation of a scene.

## Introduction

Intuitively, our visual representation of the outside world appears to be highly detailed, however, the change blindness paradigm highlights limitations of this visual representation (Rensink, 2000; Martin et al., 2001). The change blindness paradigm reveals our inability to see changes in two sequentially presented visual scenes when separated by a disruption like a saccade or a flicker (for review see: Rensink, 2002; Simons and Ambinder, 2005). Without the disruption, the change is often easy to detect. The disruption highlights the limits of our ability to retain and compare information from one visual scene to the other (Simons and Rensink, 2005).

Some changes in the visual scene are easier to detect than others. Many change blindness studies focus on the notion that changes made in parts of a scene that represent the general interpretation of the image are detected faster (Rensink et al., 1997; O’Regan et al., 1999; Shore and Klein, 2000; Stirk and Underwood, 2007; Sampanes et al., 2008). Some studies refer to the general interpretation as regions of high interest (Rensink et al., 1997; O’Regan et al., 1999; Shore and Klein, 2000; Verma and McOwan, 2010), where others refer to a semantic summary of the scene (gist) that is related to our knowledge of the world (Stirk and Underwood, 2007; Sampanes et al., 2008).

What causes these changes to be detected faster? In change blindness, subjects have to compare two image representations. Working memory is supposed to play an important role in this comparison process. One interpretation is that parts of a scene related to the general interpretation receive more attention, and are therefore more likely to be encoded and compared (Simons and Rensink, 2005). On the other hand, we know that the early visual system, i.e., primary visual cortex, encodes stimulus-driven image properties such as contrast energy (De Valois and De Valois, 1988; Boynton et al., 1999; Olman et al., 2004; Mante and Carandini, 2005; Dumoulin et al., 2008).

Some studies controlled for stimulus-driven image properties, such as stimulus, size, brightness, color, and saliency (Rensink et al., 1997; Kelley et al., 2003). These studies demonstrate that the effect of change blindness survives matching stimulus-driven properties. Yet, none of these studies considered contrast energy, the most well known computation of the early visual system. Contrast energy is not the same as saliency. Whereas contrast energy is a component of saliency, saliency is influenced by many other stimulus-driven properties. Previous change blindness studies did not always account for differences in stimulus-driven image properties (Verma and McOwan, 2010) and stimulus-driven properties might contribute to change detection, such as saliency or size of the change (Landman et al., 2003; Verma and McOwan, 2010; Spotorno et al., 2013). We predict that if change detection is founded on computations of the early visual system, contrast energy would contribute to our ability to detect changes.

Here, we ask whether contrast energy contributes to our ability to detect changes in a visual scene. We focus on contrast energy because this is one of the most well-described and most fundamental computations in early visual cortex that lies at the heart of visual computations (De Valois and De Valois, 1988). Furthermore, we compared change detection for these stimulus-driven image properties with the subjective image interpretation. Last, we investigated whether our subjective image interpretation interacts with the stimulus-driven image property contrast energy in our ability to detect changes to a visual scene.

**FIGURE 1:**
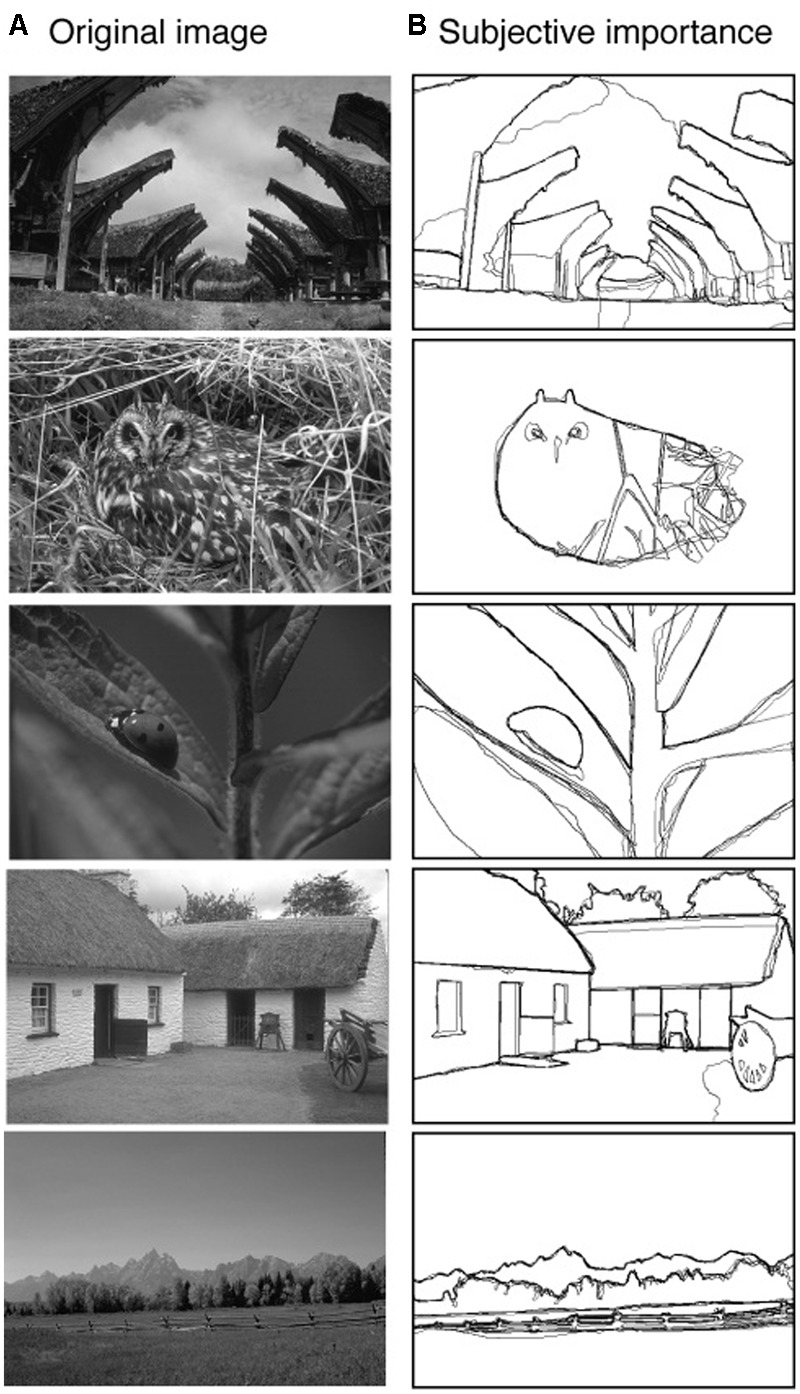
Five example images **(A)** from the ‘Berkeley Segmentation Dataset and Benchmark’ database (Martin et al., 2001). For all the images of this database human observers manually identified the important aspects of the image **(B)**. Different observers drew the labels, and their task was to draw lines on the image to highlight the parts of the image they considered to be important for the representation of the scene. We used the average manually labeled images of five observers as our definition of the subjective importance of the image. The pixels of the manually labeled images have values between 0 (not labeled) and 1 (pixel labeled by all 5 observers).

To this aim, we measured reaction times (RTs) in a flicker-task using the change-blindness paradigm (Rensink et al., 1997); subjects indicated when and where they detected the change between the images. We used images from the Berkeley Segmentation Dataset and Benchmark database (Martin et al., 2001). In this dataset, Martin et al. (2001) asked human observers to identify the most important aspects of the image (**Figure 1B**). We used these manually labeled aspects of the scene to define and quantify our measure for the subjective image interpretation. Using the combination of the natural images together with their manually labeled images, we were able to measure the amount of change that a manipulation to an image brings both to the stimulus-driven and to the subjective image interpretation. We manipulated the amount of change in subjective importance (manually labeled aspects of the scene) and in the change of the stimulus-driven image property contrast energy.

We found both significantly shorter RTs for manipulations dominated by a change in subjective importance, as well as those dominated by changes in contrast energy. Thus both subjective importance and contrast energy contribute to the speed of change detection. Furthermore, these two properties interacted: shortest RTs were found when the changes both contained a large change in contrast energy and in subjective importance. We show that this interaction effect is not explained by statistical facilitation alone, i.e., the faster RTs in the high-contrast/high-subjective importance condition are not explained by the added benefit of the presence of two independent properties (both contrast energy and subjective importance). These results show that our ability to detect changes in a visual scene is not only influenced by the high-level image interpretation of the image, but is also influenced by stimulus-driven image statistics such as contrast energy. Finally, our results that the interaction effect cannot be explained by statistical facilitation alone, suggest that the stimulus-driven and subjectively important image properties are not independently processed in the visual system, but interact with each other.

## Materials and Methods

### Participants

In total, 60 subjects participated in the experiment (30 female, age range = [18–39], mean age = 23.9, *SD* = 4.0). The total number of 60 subjects was based on a power analysis informed by previous literature (Verma and McOwan, 2010). All subjects had normal or corrected-to-normal visual acuity. The study was approved by the local Ethics Committee of the Utrecht University and the experiments were carried out in accordance with the Code of Ethics of the World Medical Association (Declaration of Helsinki). All experiments were performed with the informed written consent of the subjects. The duration of the experiment was approximately 30 min. The subjects participated either for course credits or a monetary reward.

### Apparatus

The experiment was programmed in MatLab (MathWorks, United States), using the Psychophysics Toolbox (Brainard, 1997; Pelli, 1997). Stimuli were presented on a 21-inch CRT monitor type LaCie-C22BW711 (60 Hz, 1024 × 768) using a Mac Pro 4.1 computer. The monitor was calibrated with a light meter type Gossen Mavo-Monitor USB. The viewing distance was 57 cm, which was maintained using a chin and forehead rest.

### Stimuli

**FIGURE 2:**
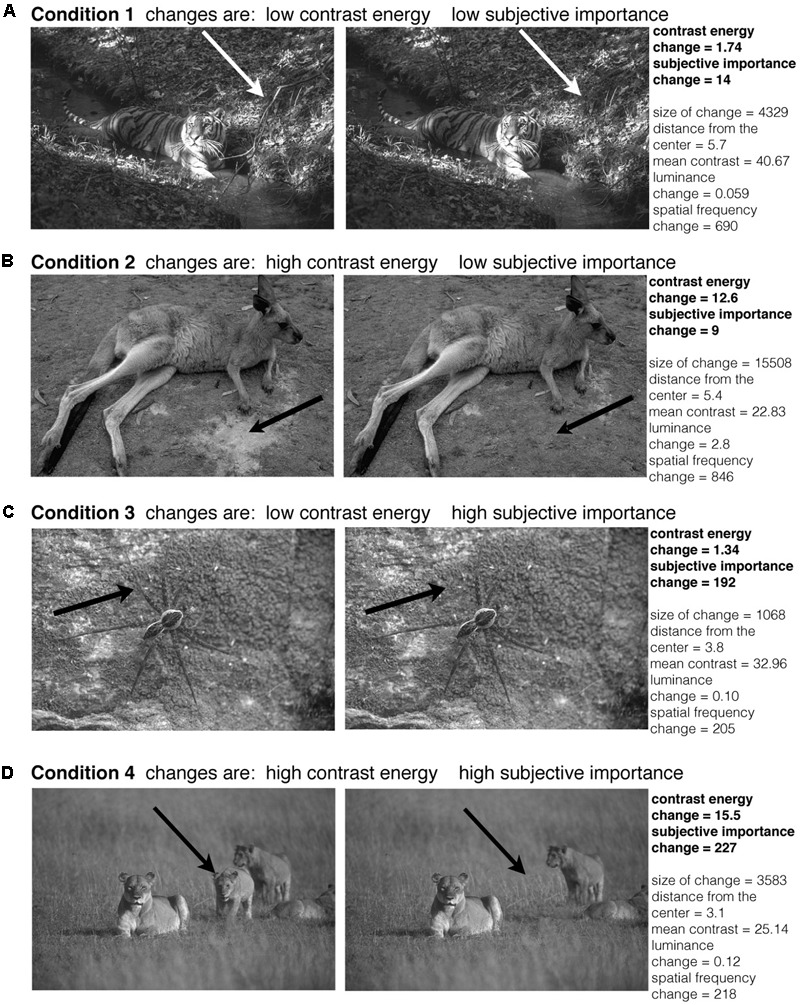
Examples of image pairs that were used in the experiment. The original images were taken from the ‘Berkeley Segmentation Dataset and Benchmark’ database (Martin et al., 2001). The arrows indicate where the manipulation in the image was made (the arrows were not present in the actual experiment). For every condition we show one example image pair **(A–D)**. The conditions are based on the amount of change in contrast energy and subjective importance. The conditions were balanced for changes in size, distance from the center, contrast, luminance and spatial frequency.

The images were taken from the ‘Berkeley Segmentation Dataset and Benchmark’ database (Martin et al., 2001). We used a selection of the grayscale images and we only used the images that were in landscape orientation. Also, we did not include any images where a human was present in the image. Using Adobe Photoshop CS6 (Version 13.0, United States), we manipulated the image content. We made a large set (502) of different changes to the images, of which we selected 108 different changes, giving 108 independent image pairs in total to be used in the experiment. To balance the different conditions, these 108 image pairs were selected from the larger set of image pairs. These 108 images were selected so every condition contained the same number of images and the changes were not made in the central region of the image; no changes were made to pixels with an eccentricity smaller than 1.6° of visual angle. Furthermore, we selected the images so there were no significant differences between the four categories in terms of eccentricity of the changes, changes in size, distance from the center, mean contrast, mean luminance change and spatial frequency change of the manipulated area. Also, multiple changes were made to the same image, but every subject only saw one change associated with one image. Changes to the images were for instance deletions of (part of) objects, or changes only in textures. **Figure 2** shows four example image pairs. The arrows indicate where the manipulation to the image was made. All stimuli were presented on a gray background. The size of the images was 481 pixels × 321 pixels, extending 18.6° × 12.4° of visual angle.

### Measure of Change in Contrast Energy and Subjective Importance

The aim of the study was to investigate the role of the stimulus-driven image property contrast energy and the subjective image interpretation on our ability to detect changes in a visual scene. We simultaneously measured the influence of both; for every image manipulation we calculated how big the change in contrast energy was and its corresponding change in subjective importance. Where we investigated the individual role of both properties, this also allowed us to measure the interaction effect of contrast energy and subjective importance on change detection.

#### Calculation of Change in the Stimulus-Driven Image Property Contrast

To compute the amount of contrast energy change that the manipulation to an image brings, the first step was to calculate the local contrast energy values, both of the original image and of the manipulated image. For every pixel of the image we calculated the local contrast energy inside a spatial window of neighboring pixels. This spatial window is defined by a Gaussian weighting function:

(1) $$w_{i}=\exp-\left(\frac{{\left({\text{x}}_{\text{i}}-{\text{x}}_{\text{c}}\right)}^{2}+{\left({\text{y}}_{\text{i}}-{\text{y}}_{\text{c}}\right)}^{2}}{2{\left(\sigma \right)}^{2}}\right)(1)$$

where (x_i_, y_i_) is the location of the *i*th pixel and (x_c_, y_c_) is the location of the pixel at the center of the image patch. σ is the standard deviation of the Gaussian window and defines the size of the spatial window. For the size of σ, we used 19.4 pixels, which corresponds to 0.75° of visual angle.

The local contrast energy value is based on the Root-Mean-Squared (RMS)-contrast (Pelli, 1997; Bex and Makous, 2002) which is defined as the standard deviation of the luminance intensities relative to the mean. The RMS-contrast is weighted by the Gaussian function to obtain the local contrast energy value per pixel:

(2) $$\text{local}\_\text{contrast}\_\text{energy}=\sqrt{\frac{1}{\sum_{\text{i}=1}^{\text{N}}w_{\text{i}}}\sum_{\text{i}=1}^{\text{N}}w_{\text{i}}\frac{{\left({\text{L}}_{\text{i}}-\text{L}\right)}^{2}}{{\text{L}}^{2}}}(2)$$

Where *w*_i_ is the Gaussian weighting function. N is the number of pixels in the spatial window. L is the mean luminance from the pixels inside the spatial window, and L_i_ is the luminance of the *i*th pixel.

**FIGURE 3:**
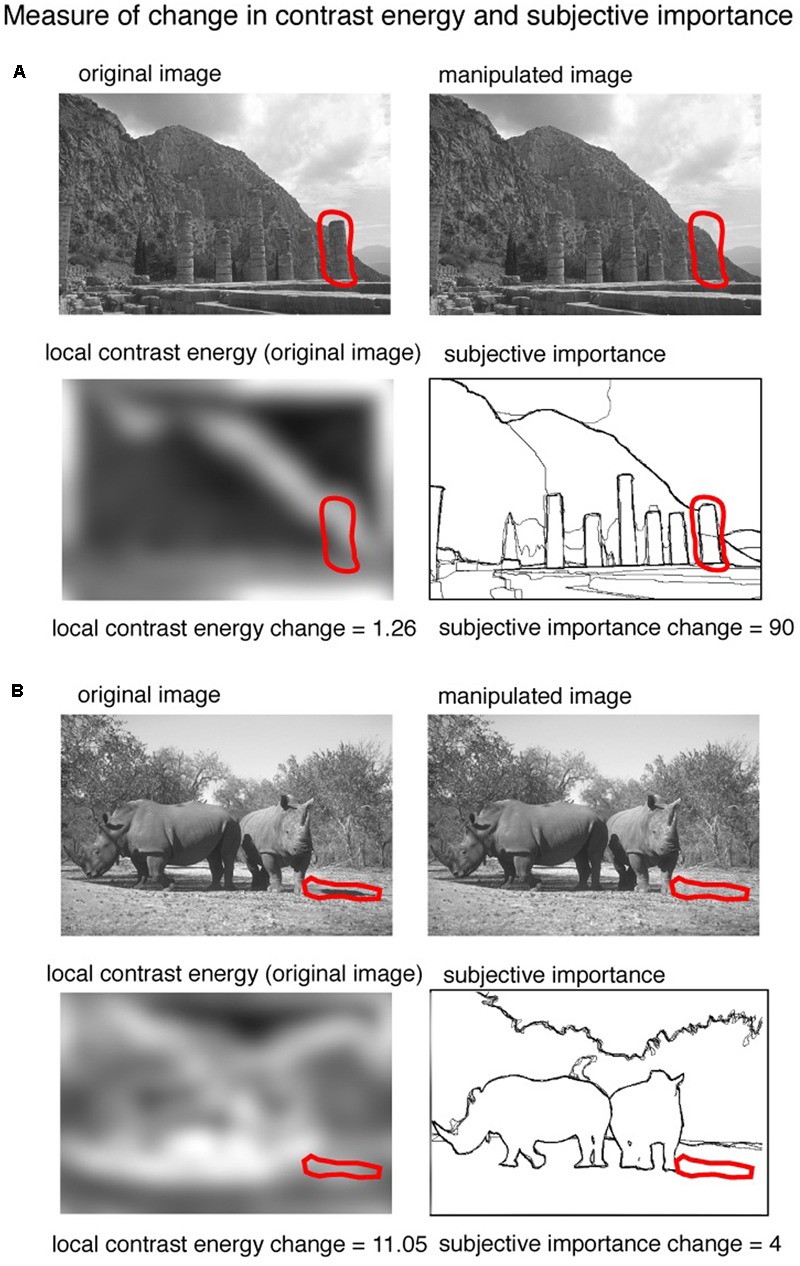
An illustration of the procedure of calculating the change in contrast energy and subjective importance. Two example images [taken from the ‘Berkeley Segmentation Dataset and Benchmark’ database (Martin et al., 2001)] are shown with changes predominant in subjective importance **(A)** and contrast energy **(B)**. The red line represents the region manipulated to alter the image. From this region we calculated the difference in local contrast energy as well as the amount of subjective importance.

Using the local contrast energy values per pixel, we computed for every image pair the change in contrast energy between the original image and the manipulated image. **Figure 3** gives an illustration of this procedure. To compute the amount of local contrast energy change, we used the local contrast energy values of the pixels that were changed in the image; the red line represents this region. The difference between the mean local contrast energy values from the original image and the mean local contrast energy values from the manipulated image defined our measure of contrast energy change.

#### Calculation of Change in Subjective Importance of the Scene

For every image from the ‘Berkeley Segmentation Dataset and Benchmark’ database (Martin et al., 2001), there are manually labeled images available. In each manually labeled image, a human observer drew lines on the image to highlight the parts of the image they considered to be important for the representation of the scene. We used the average manual labeled image of 5 observers as our definition of subjective importance. In **Figure 1** five examples of natural images (**Figure 1A**) are shown together with the averaged manually labeled images (**Figure 1B**).

We used the averaged manually labeled images to calculate the amount of change in subjective importance that a manipulation brings to an image. The pixels of the manually labeled images have values between 0 (not labeled) and 1 (pixel labeled by all 5 observers). The amount of change in subjective importance was calculated by summing the values of the pixels in the averaged manually labeled images that were manipulated. The computation is illustrated in **Figure 3**. The red line in **Figure 3** represents the changed region. From this region we computed how big the change in the manually labeled aspects of the image was and this gives us our measure of change in subjective importance from this region we also computed the amount of change in contrast energy. Two example images are shown: one in which the change is dominated by subjective importance (**Figure 3A**) and one in which the change is dominated by contrast energy (**Figure 3B**). The region was controlled for changes in size, distance from the center, mean luminance and mean contrast of the changed area and spatial frequency (see below).

### Conditions

**FIGURE 4:**
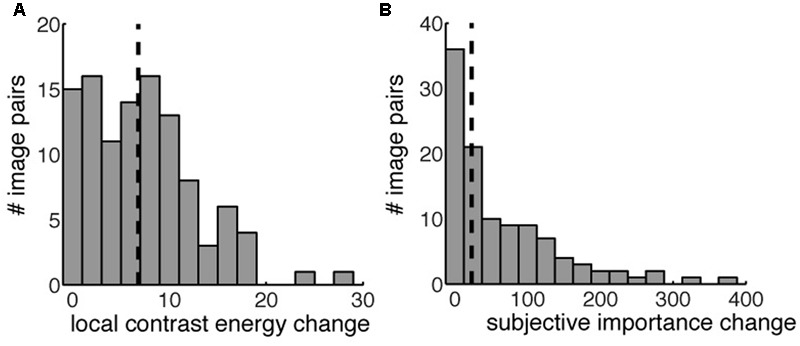
The distribution of local contrast energy change **(A)** and change in subjective importance **(B)** for all the image pairs. The vertical striped black line indicates the 50th percentile used to define our four different conditions. Image pairs left of the 50th percentile were used in the ‘low’ condition, both for contrast energy change and subjective importance change. Image pairs right to the 50th percentile were used in the ‘high’ conditions.

Based on the changes in contrast energy and subjective importance we defined four conditions based on the image-pairs that are ‘low’ and ‘high’ in their differences for contrast and subjective importance. The 108 image-pairs that we used in the experiment were selected from a larger subset of images to equally balance the four conditions. We balanced the conditions for (i) the number of images per condition and (ii) changes in size, distance from the center, mean contrast, mean luminance change and spatial frequency change of the manipulated area. We made a within subject design that compared high versus low contrast energy and high versus low subjective importance. Furthermore we also modeled the interaction between contrast energy and subjective importance. The definition of ‘low’ and ‘high’ was based on the 50th percentile of the values for contrast energy change and subjective importance change (median split). We took the first 50th percentile of the changes for the condition ‘low’ and the second 50th percentile for the condition ‘high,’ both for contrast energy as for subjective importance. **Figure 4** shows the histograms of the changes in contrast energy (**Figure 4A**) and subjective importance (**Figure 4B**); the vertical black striped line represents the 50th percentile. All the images below this boundary-line are placed in the ‘low’ condition. The images above the 50th percentile are placed in the ‘high’ condition. **Figure 2** shows for every condition an example of an image pair.

The conditions were balanced for changes in size, distance from the center, mean contrast, mean luminance change and spatial frequency change of the manipulated area. The size of the change was calculated as the number of pixels that were changed in the original image. The distance from the center was calculated as the eccentricity where the center of mass of the change was. The mean contrast was calculated as the average contrast of the manipulated area in the original image and the manipulated image. The luminance change was the difference of the mean luminance of the original image and the manipulated image in the area that was changed. The change in spatial frequency was calculated by the median of the subtracted spatial frequency distributions (calculated by fast Fourier analysis of the local image patch defined by the changed area) in the original and the manipulated image. In **Figure 2** the corresponding values for the calculated changes are reported next to the image pairs. No significant changes were found using an Anova to test for group differences [changes in size: *F*(3,104) = 0.66, *p* = 0.58, distance from the center: *F*(3,104) = 1.75, *p* = 0.16, mean contrast: *F*(3,104) = 1.17, *p* = 0.33, maximum contrast of both images *F*(3,104) = 1.07, *p* = 0.37, minimum contrast of both images *F*(3,104) = 0.60, *p* = 0.61, mean luminance difference: *F*(3,104) = 1.03, *p* = 0.38, spatial frequency difference *F*(3,104) = 0.31, *p* = 0.81], or the Kruskal–Wallis test to test for group differences [changes in size: *H*(3,104) = 2.76, *p* = 0.43, distance from the center: *H*(3,104) = 5.01, *p* = 0.17, mean contrast: *H*(3,104) = 4.18, *p* = 0.24, maximum contrast of both images *H*(3,104) = 4.51, *p* = 0.21, minimum contrast of both images *H*(3,104) = 1.84, *p* = 0.61, mean luminance difference: *H*(3,104) = 4.19, *p* = 0.24, spatial frequency difference *H*(3,104) = 1.5, *p* = 0.68].

We selected 108 different image pairs (i.e., 108 different manipulations) from a larger subset of image pairs so that every condition contained 27 of these image pairs. Some of the images were used more than once, so different manipulations to the images could be made. To make sure that every subject did not see multiple manipulations to the same image, we made 6 different subsets, in which every original image was only inserted once. The subsets contained 36 different image pairs and the subsets were balanced so that every condition contained 9 individual image pairs. Every subset of images was presented to 10 subjects. Apart from these image pairs that were used for the experiment, we also used 4 trial image pairs. Every trial image pair was an example from one of the 4 conditions. These trial image pairs were used before the actual experiment began.

### Design

For the change blindness experiment we used a flicker task (Rensink et al., 1997). In the flicker task, an original image was repeatedly alternated with a manipulated image, until the observer noticed the change. The original and the manipulated image were presented for 600 ms and were separated by a gray screen that was presented for 100 ms. The separation with a gray screen of approximately 80 ms prevents the observer from seeing the change due to movement transients caused by the change between the two images (Rensink et al., 1997). The maximum duration of the experiment was 240 s. Subjects were instructed to indicate when they saw the change by pressing the space bar. After this response, the last presented image from the image pair was presented again. At this image, the subjects indicated with the mouse where they had noticed the change in the image. After every trial the subject received feedback whether the change was correctly detected. The answer was classified correctly when the subject pointed within a small region (approximately 0.75° of visual angle) of the change. The 36 different image pairs were presented in a randomized order for every individual experiment. The experiment started with four trial image pairs to make the participants acquainted with the experiment, these trials were excluded from the analysis.

### Analysis

**FIGURE 5:**
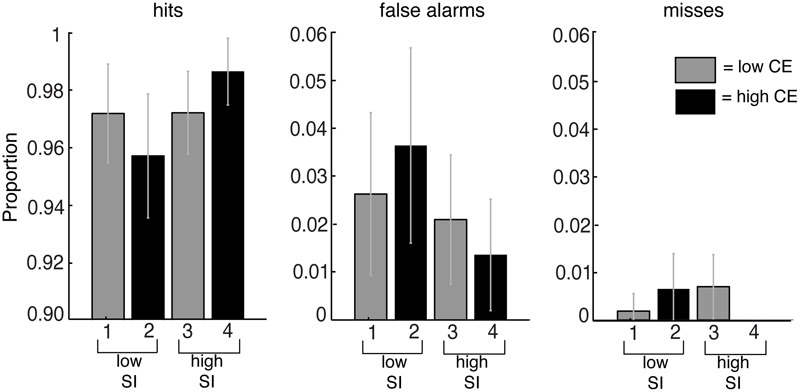
Proportion of detections (hits), detections in which subjects failed to indicate the correct location (false alarms) and the failure to detect the change (misses) for the conditions based on contrast energy (CE) and subjective importance (SI). The averaged data are the mean from all subjects, and the error bars reflect the 95% confidence interval. No significant differences were found.

We measured the time from the onset of the stimulus to the time at which the subject pressed the spacebar as the RT. Trials in which the subject did not report to see a change (misses) or did not report the change at the accurate location (false alarms) were analyzed using repeated measures ANOVA (**Figure 5**). There were no significant differences in the proportion of false alarms [*F*(3,236) = 1.39, *p* = 0.25] and misses [*F*(3,236) = 1.62, *p* = 0.19] in the different conditions, so these trials were excluded from the analysis (61 out of 2160 trials).

We analyzed the RTs using a general linear model (GLM, Matlab, Mathworks, United States). We used the GLM-approach for the analysis on the RTs, since the distributions of the RTs are skewed (non-normal). The non-normal distribution of the RTs was confirmed using a Kolmogorov–Smirnov test [*D*(2098) = 0.87, *p* << 0.001]. The GLM-approach allows the use of a non-linear linking function to compensate for the skewed RT-distributions. We used an inverse Gaussian as a linking function in the GLM. The linking function transformed the predicted variables of the GLM-model to the non-normally distribution of the RTs. Using the GLM approach we investigated the relation of the measured RTs with contrast energy change, subjective importance change and the interaction between the two using separate regressors.

We further examined whether the interaction effect can be explained by statistical facilitation alone, i.e., RTs can be faster only because more than one property (contrast energy or subjective importance) was present. For this, we compared the race-model, which is the cumulative distribution functions (CDF) of the high-contrast/low-subjective importance (RT_1_) and low-contrast/high-subjective importance (RT_2_) conditions with the CDF of the high-contrast/high-subjective importance (RT_1&2_) condition. The upper bound of statistical facilitation was calculated using the race model inequality (Raab, 1962; Miller, 1982, 1986; Ulrich et al., 2007):

(3) $$P({\text{RT}}_{1\&2}<\text{t)}\leq P\left({\text{RT}}_{1}<\text{t}\right)+P\left({\text{RT}}_{\text{2}}<\text{t}\right)(3)$$

The race model inequality reflects the probability (*P*) that an RT of the two-properties-condition (high-contrast/high-subjective importance) is equal or less to the probability of the cumulative RT distributions of the single-properties-conditions (low-contrast/high-subjective importance and high-contrast/low-subjective importance) at a given time *t*. Shorter RTs for the condition high-contrast/high-subjective importance compared to the race-model reflect a violation of the race-model and show that the interaction effect cannot be explained by statistical facilitation alone. Note that the race-model only provides an upper bound of statistical facilitation, since the CDF of the race-model sums to 2, whereas the CDFs of the single-properties-conditions only sum to 1. Therefore, only positive violations from the race-model are of interest.

Besides comparing the median RT difference between the high-contrast/high-subjective importance and the race-model, we also showed how the CDFs vary over different quantiles of the RTs (10, 20, and up to 90 percentile). Comparing the RTs for different quantiles showed the absolute difference in RTs between the condition high-contrast/high-subjective importance and the race-model at comparable points of the CDF. We bootstrapped the CDFs over our subjects (using random samples of subjects) to obtain the 95% confidence interval and the *p*-values to test for a statistical violation of the race model for the different quantiles. We corrected for multiple comparisons (number of tests = 9) using the Bonferroni method (corrected *p*-value = 0.05/9).

## Results

We measured detection rate and RTs for the four different conditions based on changes that are either low or high in contrast energy (CE) and low or high in subjective importance (SI).

The average detection rate for all changes in all image conditions was 97%, i.e., all the changes to these images were detected within the 240 s that the images were presented. **Figure 5** shows the proportion of detections (hits), detections in which subjects failed to indicate the correct location (false alarms) and failures to detect the change (misses). Since there were no significant differences in the proportion of false alarms and misses in the different conditions we excluded these trials from the analysis. Thus only the trials in which the subjects correctly detected the changes to the images were used for further analysis.

**FIGURE 6:**
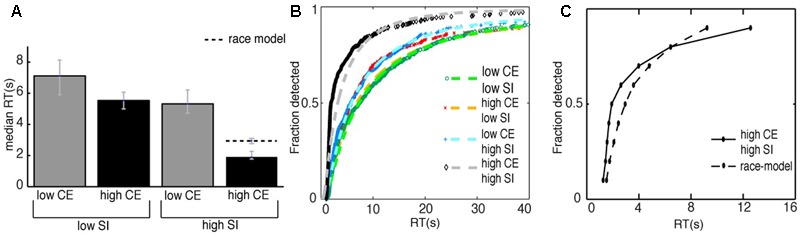
**(A)** The median reaction times of all the correct detections for all subjects for the different image conditions. The striped line represents the median RT of the race-model. The error bars reflect the bootstrapped 95% confidence interval. **(B)** The cumulative distributions of the correct responses for the different conditions. We analyzed our results using a GLM approach, with the inverse Gaussian as a linking function. The striped lines are the fits to the data with an inverse Gaussian function. Note that we only show the RTs up to the first 40 s of the experiment here, the entire RT-range is 0–240 s. We found significantly shorter reaction times for both changes in contrast energy (CE) and subjective importance (SI). Furthermore, we found a significant interaction effect, i.e., reaction times were shorter when changes affected both contrast energy and subjective importance. **(C)** The comparison of the CDF of the condition high-contrast/high-subjective importance with the CDF of the race-model. The CDF of the race-model is the summed CDFs of the conditions high-contrast/low-subjective importance and low-contrast/high-subjective importance. We found statistical significant shorter RTs for the condition high-contrast/high-subjective importance compared to the race-model for the 10th to the 70th percentile. This indicates that the interaction effect cannot be explained by statistical facilitation alone.

We measured the time from the beginning of the stimulus, until the time the subjects noticed the change and pressed the spacebar as the RT. **Figure 6A** shows the median RTs per condition, the error bars reflect the bootstrapped 95% confidence intervals. The condition in which the manipulations to the image were the most difficult to detect was the low-contrast/low-subjective importance condition, i.e., in this condition the RT was longest (median RT = 7.1 s). Comparing this to the high-contrast/low-subjective importance condition, we see that changes in the latter condition were detected faster (median RT = 5.5 s, median decrease RT = 1.6 s). Comparing the first condition to the low-contrast/high-subjective importance condition, we also found a faster detection of the manipulations (median RT = 5.3 s, median decrease RT = 1.8 s). Last, the manipulations of the high-contrast/high-subjective importance condition were detected fastest (median RT = 1.9 s, median decrease RT = 5.2 s). These results indicate an effect both for the amount of subjective importance change and the amount of contrast energy change on the RT. A manipulation to an image with a larger change in either contrast energy or subjective importance to the image led to a faster detection of the manipulation. The results also suggest an interaction effect for the amount of contrast energy change and the amount of subjective importance change. A manipulation that was both high in its change for contrast energy and subjective importance resulted in the fastest detection of the manipulation.

To quantify these observations, we performed a GLM-analysis to compare the effects of contrast energy change, subjective importance change and the interaction of the two on the RTs. Since the distribution of RTs was skewed, we did not use the canonical linking function that tests for differences in the conditions based on their mean. Instead of the canonical linking function, we used the inverse Gaussian linking function to take into account the non-normal or non-Gaussian distribution of the data. The differences of the distributions are now based on the fit of the inverse Gaussian distribution. **Figure 6B** shows the fits of the inverse Gaussian function to the distribution of the data for the different conditions. All fits had a variance explained *R*^2^ > 0.97. Using the GLM analysis, we found a significant effect for the level of contrast energy change: *t*(2095) = 9.92, *p* < < 0.001. Where the number 2095 reflects the total amount of (correct) RTs from all the subjects. Also, we found a significant effect for the level of subjective importance change: *t*(2095) = 6.54, *p* << 0.001. Furthermore, we found a significant effect for the interaction of the amount of contrast energy change and the amount of subjective importance change: *t*(2095) = 7.90, *p* << 0.001. Thus, our ability to detect changes in a visual scene is influenced by both the subjective image interpretation and the stimulus-driven image properties, and they interact.

Besides our analysis based on the four different conditions, we repeated the analysis using a GLM with ranked values for contrast energy and subjective importance. Using this analysis we found a significant effect for both contrast energy (*p* < 0.001) and the interaction of contrast energy and subjective importance (*p* = 0.013). However, the effect for subjective importance was not significant (*p* = 0.0578). This analysis assumes a linear relationship between the values for contrast energy and subjective importance, which is not likely the case and we assume this is the reason why we don’t find a significant effect for subjective importance using this analysis method. Therefore, we believe that the split analysis is more correct.

To further examine this interaction effect, we investigated whether it can be explained by statistical facilitation alone. We compared the CDF of the high-contrast/high-subjective importance condition to the CDF of the race model (**Figure 6C**). We compared the bootstrapped CDFs at different quantiles, from the 10th to the 90th percentile. For the 10th to the 70th percentile we found a statistically significant violation of the race model (*p* < 0.001 for all significant percentiles, Bonferroni corrected for multiple comparisons), with a maximum of 1 s difference in RT that was unexplained by statistical facilitation.

## Discussion

In our study we used manually labeled images to investigate the simultaneous effect of stimulus-driven and subjectively important image properties on our ability to detect changes in a visual scene. We measured RTs within a change blindness task and compared RTs over four different conditions. The conditions were based on the amount of change in the stimulus-driven image property contrast energy and the amount of change in the subjective image interpretation defined by the manual labels.

### Shorter RTs for High Change in Subjective Importance

Analyzing the RTs for the different conditions, first, we found a significant effect for subjective importance. RTs were shorter for manipulations that have a larger change in their subjective image interpretation. This measure of change was based on the manually labeled aspects of the images, where people indicated which parts represented the image most (Martin et al., 2001). Our results are in line with previous change blindness studies, which showed that changes made in parts of a scene that were considered to be important for their high-level image representation were detected earlier (Rensink et al., 1997; O’Regan et al., 1999; Shore and Klein, 2000; Stirk and Underwood, 2007; Sampanes et al., 2008; Verma and McOwan, 2010). However, different notions of the high-level image representation have been used in these studies. Some refer to it as the gist; the general interpretation of a scene (Stirk and Underwood, 2007; Sampanes et al., 2008), where others refer to it as regions of high interest (Rensink et al., 1997; O’Regan et al., 1999; Shore and Klein, 2000; Verma and McOwan, 2010). Our study shows most resemblance to the studies that use the notion of the high-level image interpretation as regions of high interest.

### Shorter RTs for High Contrast Energy Change

We also found a significant effect for the amount of contrast energy change on the RTs. Manipulations with a larger change in contrast energy were detected earlier. Neurophysiological data shows that the early visual cortex responds strongly to changes in contrast energy (De Valois and De Valois, 1988; Boynton et al., 1999; Olman et al., 2004; Mante and Carandini, 2005; Dumoulin et al., 2008). We speculate that this preference of early visual cortex underlies the ability to detect these changes. Alternatively, changes in contrast energy might attract more (exogenous) attention. These two explanations are not mutually exclusive and both or either one may explain why changes in contrast might be detected earlier.

Most change blindness studies do not account for effects of stimulus-driven image statistics. Some studies did, but show deviating results. Verma and McOwan (2010) and Spotorno et al. (2013) found faster detection for changes in a region with high salience whereas, Stirk and Underwood (2007) did not. Contrast energy is not the same as saliency. Depending on the definition, saliency is a complex combination of image features such as variations in color, luminance and orientation (Itti and Koch, 2000; Borji et al., 2013). Thus, depending on the definition, saliency will involve contrast-energy but also many other stimulus-driven features. We focused on contrast energy as it is well defined and a well-known computation of early visual cortex. We show that contrast energy can lead to differences in the detection of changes. This opens the door to investigate other image properties and computations of the early visual system that may potentially also contribute to change blindness, such as orientation and spatial frequency (Hubel, 1982), spatial coherence (Groen et al., 2012, 2013), and contour integration (Field et al., 1993).

### Interaction of Change in Contrast Energy and Subjective Importance

Last, we found a significant interaction effect for the amount of change in subjective importance and contrast energy change. By using the combination of the manually labeled images simultaneously with the inherent contrast energy of the images, we were able to investigate not only the effect of stimulus-driven and subjectively important image properties on the change blindness paradigm. Most importantly, we were able to simultaneously measure the effect of both because the levels of contrast change and change in subjective importance were independent in the images.

The interaction we find cannot be explained by the added benefit of two independent observations (race-model). This suggests that the stimulus-driven and subjectively important image properties are not independently processed in the visual system, but instead they facilitate each other. Perception does not only rely on visual input but also on our knowledge of the world (von Helmholtz, 1867). Contrast-energy is solely determined by the visual input, whereas both visual input and our knowledge of the world drive the subjective importance judgments. Therefore, we speculate that this interaction suggests that, like perception, change blindness also relies on visual input and our knowledge of the world.

This interaction between contrast energy and subjective importance may be implemented in different ways in the visual system. For example, different theories propose that higher-order areas feedback to lower-order areas and modulate their responses according to the prior expectations about the visual world (Mumford, 1992; Koch and Poggio, 1999; Rao and Ballard, 1999; Friston, 2005). We speculate that this neural interaction is the origin of the behavioral interaction effect of the subjectively important image representation and the stimulus-driven image statistics.

## Conclusion

While earlier studies on change blindness conclude that our ability to detect changes in a visual scene is influenced by the subjective image interpretation of the scene, we find that this ability is also influenced by the stimulus-driven image property contrast energy. Specifically, large changes in subjective importance and contrast energy are both detected faster than small changes in these properties. Moreover, these two properties interact; image manipulations that strongly affect both subjective importance and contrast energy are detected fastest, and importantly, faster than expected based on the amount of change of either property alone. We also found an interaction effect; manipulations that were both high in their change of subjective importance and contrast energy were detected earliest. Presumably, these parts of an image are processed in greater detail. The change blindness paradigm can be used to give more insight in how stimulus-driven image statistics and subjective image representations are processed in the visual system. Our results suggest that they are not processed independently, but instead interact in how they are represented in the visual system.

## Author Contributions

WZ and SD designed the study. All authors contributed to the study design. Stimulus creation and data collection was performed by WZ and JvL. WZ performed the data analysis and interpretation under the supervision of SD. WZ drafted the manuscript, and JvL and SD provided critical revisions. All authors approved the final version of the manuscript for submission.

## Conflict of Interest Statement

The authors declare that the research was conducted in the absence of any commercial or financial relationships that could be construed as a potential conflict of interest.
